# Predicting transitions between functional and financial vulnerability states and death in older Chinese adults: a longitudinal CHARLS study with temporal evaluation

**DOI:** 10.21203/rs.3.rs-10694017/v1

**Published:** 2026-09-29

**Authors:** Tianrun Piao, Aiqun He, Jingbo Li

**Affiliations:** Guangdong Work Injury Rehabilitation Hospital; Guangdong Work Injury Rehabilitation Hospital; Guangdong Work Injury Rehabilitation Hospital

**Keywords:** activities of daily living, catastrophic health expenditure, older adults, multistate transitions, prediction, temporal evaluation, CHARLS

## Abstract

**Background:**

Functional decline can increase care needs at the same time that out-of-pocket spending constrains continued treatment and rehabilitation. Existing studies usually predict these outcomes separately and therefore cannot identify older adults moving into joint functional and financial vulnerability. We developed a multistate model and tested whether its predictions remained informative in later CHARLS waves.

**Methods:**

We used 2011–2018 Harmonized China Health and Retirement Longitudinal Study data. Adults aged at least 60 years contributed adjacent-wave observations. Four living states combined basic-activity difficulty and household catastrophic health expenditure, defined as out-of-pocket health spending at least 40% of non-food expenditure; death was the fifth state. Penalized multinomial regression and XGBoost were developed in 2011–2015 and evaluated without updating in 2015–2018. Penalized regression was primary because it matched XGBoost while remaining transparent. The locked model was applied to 2018 origins for exploratory 2020 validation of observable death and ADL components.

**Results:**

The analysis comprised 12,866 transitions from 7,353 participants; 5,175 formed the 2015–2018 evaluation set, including 361 dual-vulnerability outcomes and 389 deaths. Penalized regression achieved a macro-AUROC of 0.722 (95% CI 0.712–0.734) and Brier score of 0.570 (0.557–0.583). AUROCs were 0.728 for dual vulnerability, 0.755 for death, and 0.629 for CHE alone. XGBoost did not improve macro-AUROC (0.720). Of 8,532 eligible 2018 origins, 8,004 had death or ADL status observed in 2020. Exploratory component AUROCs were 0.813 for death and 0.801 for ADL difficulty or death. The highest-risk 20% contained 53.5% of deaths and 46.1% of dual-vulnerability-or-death outcomes.

**Conclusions:**

Joint functional-financial states were dynamic and prognostically distinct. A transparent penalized model provided moderate discrimination for dual vulnerability and death in the complete five-state evaluation and identified a manageable high-risk stratum for coordinated functional and financial review. The 2020 analysis supported only the observable clinical components. Isolated CHE remained difficult to forecast without detailed reimbursement, local-cost, and long-term-care information.

## Background

Loss of independence and inadequate financial protection are two of the most consequential threats to ageing well. Difficulty with basic activities of daily living (ADL) signals an immediate need for personal assistance, rehabilitation, or long-term care. Catastrophic health expenditure (CHE) signals that a household is devoting an exceptionally large share of disposable resources to health care. When these conditions occur together, care needs intensify just as the household’s capacity to sustain treatment, nutrition, housing, and rehabilitation contracts. Identifying this joint state before the next follow-up can therefore inform both geriatric care and financial-protection services.

Chinese evidence has described substantial CHE among older households and has linked CHE to disability, multimorbidity, health-care use, low socioeconomic position, cognitive impairment, and reduced intrinsic capacity [3–6, 9, 15, 16]. Longitudinal work has also examined trajectories of functional disability and poverty [7], health-state transitions and medical expenditure [8], and the effects of long-term care insurance on catastrophic spending [13]. These studies establish the connection between health loss and financial strain, but they do not estimate where an individual–household unit will be at the next observation when recovery, isolated functional limitation, isolated CHE, joint vulnerability, and death are all possible outcomes.

Prediction research based on CHARLS has expanded rapidly. Recent studies have developed models for incident disability, instrumental ADL limitation, and fall risk in clinically defined groups [10–12, 26]. These studies demonstrate the clinical appeal of a specific binary outcome, but most exclude participants who already have the outcome and use a random train–test split. Such designs cannot represent recovery or movement among competing functional and financial states, and random splitting does not test transport across calendar time, changing prices, insurance arrangements, or population ageing. A targeted PubMed search completed on 13 August 2026 identified no study that combined ADL difficulty, CHE, recovery, death, and prediction in a multistate framework (Additional file 1).

We therefore treated functional and financial vulnerability as a joint, reversible state process with death as an absorbing state. The objectives were to: (1) describe movement among four living states and death; (2) develop penalized multinomial regression and XGBoost models in early CHARLS transitions and evaluate them in the later 2015–2018 transition without updating; (3) use the simpler model as the primary model if predictive performance was comparable; (4) explore whether its death and ADL components retained discrimination in 2018–2020; and (5) translate predicted probabilities into capacity-based high-risk strata relevant to coordinated geriatric, rehabilitation, and financial-protection services.

## Methods

### Study design and data source

We conducted a longitudinal prediction model study using Harmonized CHARLS Version D, containing the nationally representative 2011, 2013, 2015, and 2018 waves, together with official 2020 CHARLS public-use files [1, 23, 24, 27]. CHARLS used multistage probability sampling to recruit Chinese residents aged 45 years or older and their spouses from 150 counties and 450 villages or urban communities across 28 provinces [1]. Reporting followed TRIPOD + AI [17], and methodological choices were checked against PROBAST + AI domains[18].

### Landmark cohort

Each survey wave was treated as a landmark. A participant could enter at an origin wave if interviewed, aged at least 60 years, and classifiable into one of four living states. The destination was the next regular wave: 2011–2013, 2013–2015, or 2015–2018. We retained an observation when the next living state was observed or death had been recorded. The unit of analysis was an adjacent-wave person-transition. Repeated transitions from the same participant were allowed because the task was dynamic updating, but household grouping was preserved during resampling and uncertainty estimation.

### Outcome states

Basic ADL function was measured with six items covering dressing, bathing, eating, transferring into or out of bed, toileting, and continence [2]. Any reported difficulty defined ADL difficulty. Household annual out-of-pocket spending combined the respondent’s and spouse’s inpatient spending during the previous year and outpatient spending during the previous month multiplied by 12. A missing cost was set to zero only when the corresponding service-use indicator recorded no use. Spouse costs were set to zero for respondents not married or partnered; when use was reported but cost was missing, the household expenditure outcome remained missing.

Capacity to pay was annual non-food household consumption: 12 times monthly non-food spending plus annual non-food spending. CHE was defined as out-of-pocket health spending at least 40% of capacity to pay, consistent with the widely used capacity-to-pay approach [3, 22]. Four living states were then defined: (1) no ADL difficulty and no CHE, (2) ADL difficulty only, (3) CHE only, and (4) dual vulnerability, meaning both ADL difficulty and CHE. Death at the destination wave formed state 5.

### Predictors

Predictors were measured at each origin landmark and chosen before model fitting on availability, plausibility, and consistency across waves. They were age; sex; education; marital or partnership status; rural residence; self-rated health; number of ADL difficulties; count of 12 self-reported chronic conditions; 10-item Center for Epidemiologic Studies Depression score; a composite cognitive score from immediate and delayed recall, serial sevens, orientation, and figure drawing; log-transformed household capacity to pay; household size; number of living children; public health insurance; current smoking; current drinking; social activity; weekly contact with children; hospital admission in the past year; outpatient visit in the past month; and the four-category origin state. The outcome components themselves were not included except through the origin state and ADL count; no destination information was used.

### Model development and temporal evaluation

Transitions from 2011 to 2013 and from 2013 to 2015 formed the development data. The 2015–2018 transition was locked as temporal evaluation data and was not used for hyperparameter selection, imputation, or scaling. Penalized multinomial logistic regression and XGBoost were compared using household-grouped cross-validation and log loss. Because the simpler penalized model matched or slightly exceeded XGBoost in the locked evaluation, it was selected as the primary model; XGBoost was retained as a nonlinear comparator.

Continuous predictors were median-imputed with missingness indicators and standardized. Categorical predictors were mode-imputed with missingness indicators and one-hot encoded. All preprocessing occurred within each fitted pipeline. Hyperparameters were selected in five-fold group cross-validation using household as the grouping unit and multinomial log loss as the selection criterion. The final development pipelines were then applied once to the later wave. Development out-of-fold estimates used the selected hyperparameters and are presented as internal estimates, not as a substitute for the temporal evaluation.

### Performance and interpretation

Discrimination was quantified for each destination state with one-vs-rest AUROC and AUPRC; macro averages gave equal weight to all five states. Overall probabilistic accuracy was summarized with the multiclass Brier score and log loss. Calibration was evaluated graphically and with the mean predicted probability, observed-to-expected ratio, calibration intercept, and calibration slope for each state [19]. Ninety-five percent confidence intervals were obtained from 300 bootstrap samples clustered by household. Variable-level permutation importance was the decrease in macro one-vs-rest AUROC after 20 independent permutations in the temporal data. Importance reflects predictive contribution and was not interpreted causally.

### Sensitivity analyses

We evaluated the primary model in households absent from the development data, calculated performance with CHARLS respondent sampling weights, and repeated temporal evaluation in an age-extended cohort of adults aged 45 years or older. Outcome construction was audited at CHE thresholds of 25%, 30%, 40%, and 50% of capacity to pay. We also reported temporal performance separately by sex and rural residence. Missing predictors were imputed within the model pipeline; observations without an origin or destination outcome were not imputed.

### Late temporal component validation in 2020

The primary five-state outcome could not be reconstructed in 2020 because that wave did not include outpatient and inpatient out-of-pocket components comparable with 2011–2018. We therefore conducted a secondary exploratory component-level validation that did not alter the fitted model. The locked penalized regression model was applied to all classifiable 2018 origins aged 60 years or older. Official 2020 files supplied vital status and the same six basic ADL items. We evaluated death, ADL difficulty or death, and ADL difficulty among survivors. These analyses assess transport of observable clinical components only; they do not validate CHE, dual vulnerability, the complete five-state outcome, or an independent external cohort. Interpretation also accounts for the exceptional COVID-19 period.

### Clinical interpretation and additional robustness analyses

To show the direction and practical scale of model predictions, we generated standardized model profiles by setting age, ADL difficulty count, chronic disease count, or origin state to clinically interpretable values while leaving all other observed predictors unchanged. We also evaluated fixed service-capacity scenarios in which the highest predicted-risk 10%, 20%, or 30% would receive further assessment; these scenarios were descriptive and were not optimized against the evaluation outcome. In an exploratory extension, grip strength, walking-test time, five-chair-stand time, and measured body mass index were added with in-pipeline imputation, so participants without physical tests were retained. Finally, inverse-probability-of-observation weights estimated from all valid 2015 origins were used to assess sensitivity to missing 2018 outcomes.

### Use of generative artificial intelligence

During manuscript preparation, the authors used OpenAI Codex for language editing, document formatting. The authors independently verified all analyses, numerical results, citations, interpretations, and manuscript content, revised the output critically, and take full responsibility for the article.

## Results

### Cohort and observed transitions

Across the three origin landmarks, 25,659 observations met the age and interview criteria. An origin state was available for 18,026; 12,866 also had an observed destination state or death and entered analysis (Fig. 1). These transitions came from 7,353 participants in 4,723 households. Development included 7,691 transitions and temporal evaluation 5,175. The first eligible landmark profile is shown in Table 1. Mean age was 66.4 years, 48.2% were women, 64.3% lived in rural areas, and 17.7% already had ADL difficulty without CHE, 14.0% CHE without ADL difficulty, and 6.7% dual vulnerability. Predictor missingness was at most 10.5% for cognition; all other predictors were missing in less than 8.4% of transitions (Additional file 1).

The diagram summarizes cohort construction, early-wave development, locked 2015–2018 five-state temporal evaluation, and 2018–2020 late component validation. Counts are person-landmark observations; participants could contribute more than one adjacent-wave transition. The 2020 analysis evaluated death and ADL components because comparable CHE variables were unavailable.

Values are mean (standard deviation) or n (%). The first eligible origin landmark was retained for each participant. Percentages use the full cohort denominator and may not sum to 100 because of rounding or missing data. ADL, activities of daily living; CESD-10, 10-item Center for Epidemiologic Studies Depression scale; CHE, catastrophic health expenditure.

Destination-state counts were 2,026/602/555/247/266 for no vulnerability, ADL difficulty only, CHE only, dual vulnerability, and death in 2011–2013; 2,129/723/620/290/233 in 2013–2015; and 2,818/887/720/361/389 in 2015–2018. Transitions were not monotonic (Fig. 2). Among observations starting with dual vulnerability, 24.9% moved to no ADL difficulty and no CHE, 30.6% to ADL difficulty only, 10.5% to CHE only, 19.4% remained dually vulnerable, and 14.6% ended in death. By comparison, death followed 4.7% of observations starting with neither vulnerability and 5.5% of those starting with CHE alone.

Row percentages pool adjacent-wave observations from 2011 through 2018. CHE denotes catastrophic health expenditure and ADL activities of daily living.

### Temporal model performance

In the 2015–2018 temporal evaluation, the primary penalized model achieved a macro-AUROC of 0.722 (95% CI 0.712–0.734), macroAUPRC of 0.351 (0.340–0.366), and multiclass Brier score of 0.570 (0.557–0.583) (Tables 2 and 3). AUROC was 0.728 for dual vulnerability, 0.755 for death, and 0.629 for CHE alone. XGBoost did not improve aggregate discrimination (macro-AUROC 0.720) or probabilistic accuracy (Brier score 0.569).

Temporal evaluation comprised 5,175 transitions from 2015 to 2018. AUROC and AUPRC are one-vs-rest estimates. Confidence intervals used 300 household-clustered bootstrap samples.

Macro averages give equal weight to each destination state. Lower Brier score and log loss indicate better probabilistic accuracy.

Primary-model mean predicted probabilities were 51.7% for no vulnerability, 18.4% for ADL difficulty only, 15.8% for CHE only, 8.0% for dual vulnerability, and 6.0% for death; observed proportions were 54.5%, 17.1%, 13.9%, 7.0%, and 7.5%. Calibration slopes ranged from 0.847 to 1.134 (Table 2; Fig. 3).

Points compare mean predicted probability with observed proportion within eight quantile groups for each destination state. The dashed line denotes perfect calibration.

### Predictive information and sensitivity analyses

The number of ADL difficulties produced the largest loss in macro-AUROC when permuted in the primary penalized model, followed by age, chronic disease count, origin state, marital or partnership status, depressive symptoms, self-rated health, and cognition (Fig. 4). Directional model profiles were consistent with these rankings. Holding other observed predictors fixed, predicted ADL-difficulty-or-death risk increased with ADL difficulty count, multimorbidity, and age. These are descriptions of model behavior rather than causal effects. Importance is the decrease in macro one-vs-rest AUROC after permuting one origin-landmark variable, averaged over 20 repetitions. Values describe predictive contribution, not causality.

Among 1,882 temporal observations from households absent from development, macro-AUROC was 0.723; AUROCs were 0.741 for dual vulnerability and 0.771 for death. Sampling-weighted macro-AUROC was 0.724. Inverse-probability-of-observation weighting gave a macro-AUROC of 0.723 and Brier score of 0.568. The age-extended temporal analysis produced a macro-AUROC of 0.746. Macro-AUROC was 0.722 in men and 0.719 in women, and 0.735 in urban and 0.712 in rural participants. Results across CHE thresholds are reported in Additional file 1.

### Late temporal component validation

Among 8,532 classifiable 2018 origins, vital status was available for 8,010 and death or ADL status for 8,004 (93.8%); 409 participants died and 2,300 of 7,595 observed survivors had ADL difficulty. Without refitting or recalibration, the primary model achieved AUROCs of 0.806 for death, 0.799 for ADL difficulty or death, and 0.793 for ADL difficulty among survivors. These secondary findings concern observable clinical components only and do not constitute validation of the complete five-state outcome (Table 4; Fig. 5).

The development-locked penalized multinomial model was applied without updating to 2018 origins. Confidence intervals used 300 household-clustered bootstrap samples. This secondary exploratory analysis evaluated observable death and ADL components only; the 2020 wave did not permit reconstruction of the complete CHE-based five-state outcome. AUPRC, area under the precision-recall curve; AUROC, area under the receiver operating characteristic curve; ADL, activities of daily living.

Panel A shows AUROCs with 95% household-clustered bootstrap confidence intervals for three observable outcomes. Panel B compares observed outcome frequency with mean predicted risk. The 2011–2015 penalized multinomial model was applied without refitting or recalibration; the complete five-state outcome could not be reconstructed in 2020.

### Clinical risk strata and objective physical performance

In 2015–2018, the highest predicted-risk 20% contained 208 of 389 deaths (53.5%) and 346 of 750 dual-vulnerability-or-death outcomes (46.1%). Mortality was 20.1% in this stratum versus 4.4% in the remainder; corresponding combined-outcome rates were 33.4% and 9.8% (Fig. 6). These fixed capacity scenarios illustrate service concentration and are not treatment thresholds. The small performance difference between the two algorithms supports using the transparent penalized model as the primary model and treating XGBoost as a nonlinear comparator.

Panel A shows the proportion of subsequent events captured when the highest predicted-risk 10%, 20%, or 30% received further assessment. Panel B compares event rates in the highest-risk 20% with the remaining 80%. Fractions were fixed service-capacity scenarios, not outcome-optimized treatment thresholds.

Objective physical measures were missing in 14.4%−25.0% across analysis sets but were imputed within the model pipeline. Adding grip strength, walking time, chair-stand time, and body mass index changed macro-AUROC from 0.722 to 0.724; dual-vulnerability AUROC remained 0.728 and death AUROC increased from 0.755 to 0.761. The small gain supports retaining the scalable core model while using physical tests for clinical assessment rather than requiring them for initial screening.

## Discussion

### Principal findings

This study shows that functional and financial vulnerability in later life form a dynamic joint process rather than two fixed endpoints. The primary penalized model provided moderate five-state discrimination and broadly useful calibration in 2015–2018. XGBoost offered no material gain, showing that the available signal was largely captured without a more complex algorithm. Exploratory 2020 analyses supported transport of mortality and ADL components, but not CHE or the complete five-state outcome.

The main contribution is not algorithmic novelty. Penalized regression slightly exceeded XGBoost in macro-AUROC and offered greater transparency, so it was used as the primary model. The contribution lies in defining a state process that joins care need and household affordability, preserving recovery and competing death, and evaluating the full model in a later calendar period. The poor discrimination for CHE alone sets an important boundary: routinely collected clinical and household variables cannot substitute for detailed reimbursement, benefit-design, local-price, acute-event, and transfer-payment data.

### Comparison with previous studies

Earlier CHARLS studies found that disability, multimorbidity, health-care use, and socioeconomic disadvantage increased CHE risk [4–6, 15, 16]. Work on functional disability and poverty showed that worsening functional trajectories were linked to persistent disadvantage [7], while health-state modeling linked disability severity to medical expenditure [8]. More recently, lower intrinsic capacity predicted CHE and CHE was associated with later health outcomes [9]. Our transition matrix extends this literature by locating functional limitation and CHE in a single state space and showing that their combination marks a destination pattern distinct from either component alone.

Prior CHARLS prediction studies generally examined a single binary outcome [10–12]. A recent machine-learning study of falls among 1,087 older adults with sarcopenia illustrates the clinical appeal of a narrowly defined endpoint [26]. Our task is different: given a person’s current functional-household financial state, which of five competing states is most probable next? The five-state problem retains recovery and competing death but also exposes the limited predictability of isolated CHE. The 2020 component analysis is exploratory and cannot establish long-term stability of the complete state model.

### Interpretation

Functional burden was the strongest source of predictive information. This raises the legitimate concern that discrimination for dual vulnerability partly reflects recognition of ADL limitation. Dual vulnerability nonetheless remained distinct from ADL-only because the model had to discriminate it simultaneously from four competing outcomes. The contrast between moderate dual-vulnerability discrimination and weak CHE-only discrimination should therefore be read as evidence for a recognizable high-need, high-burden joint state, not as accurate prediction of economic shocks in otherwise functionally independent households.

CHE alone was less predictable than dual vulnerability or death. Out-of-pocket spending is episodic and depends on realized service use, benefit design, reimbursement, provider choice, regional prices, and unmeasured care-seeking preferences. These factors can change faster than functional status and are only partly represented in harmonized survey predictors. The result is substantively useful: financial risk prediction cannot be solved by importing a standard geriatric risk score. Better forecasting of CHE will require more detailed insurance entitlements, reimbursement ratios, local price and supply measures, and long-term and informal care costs.

### Implications

The findings support a two-stage pathway. First, routinely available age, ADL, multimorbidity, psychosocial, health-care-use, and household information can identify a service-capacity-defined risk stratum. Second, those identified should receive a combined review rather than a single disease referral: comprehensive geriatric and rehabilitation assessment; medication, falls, nutrition, cognition, and depressive-symptom review; clarification of insurance reimbursement and medical-assistance eligibility; and assessment of long-termcare and caregiver needs. The highest-risk 20% scenario captured more than half of subsequent deaths and almost half of dualvulnerability-or-death outcomes, providing a concrete planning benchmark. It is not yet suitable for denying services or determining treatment without external validation, threshold-specific utility analysis, and prospective impact evaluation.

The subgroup audit also matters for implementation. Aggregate discrimination differed little by sex, but dual-vulnerability discrimination was lower in women, and both aggregate and mortality discrimination were lower in rural residents. These differences do not establish unfairness, but they argue against deploying a single threshold without checking local error rates and access consequences.

### Strengths and limitations

Strengths include a nationally sampled longitudinal cohort; a joint outcome retaining recovery, deterioration, and death; householdgrouped resampling; a locked 2015–2018 complete five-state temporal evaluation; explicit calibration; comparison of a transparent penalized model with a nonlinear alternative; capacity-based risk strata; and sensitivity analyses for observation weighting, survey weights, age, CHE threshold, new households, sex, and residence. The 2020 outcomes were not used to refit or select the model and were interpreted only as exploratory component-level evidence.

Several limitations define the boundary of interpretation. First, 7,633 age-eligible landmarks lacked a classifiable origin and 5,160 valid origins lacked an observed destination; the latter represents 28.6% of valid origins. Inverse-probability weighting addressed selection explained by measured origin characteristics but cannot remove bias from unmeasured causes of missingness or missing-not-at-random OOP data. Second, ADL is individual-level whereas CHE is household-level. The state therefore represents person-household vulnerability and does not attribute household spending to the indexed individual; spouses can share the same CHE exposure despite household-grouped analysis. Third, annual outpatient OOP was estimated by multiplying one-month expenditure by 12. Episodic spending, seasonality, recall error, and a large survey-month expense may respectively understate or overstate annual burden and likely contribute to poor CHE-only discrimination. Fourth, 2020 lacked comparable CHE components, so only mortality and ADL components were explored; the COVID-19 period further limits interpretation. Fifth, development and evaluation intervals differed. Finally, variable importance and standardized profiles describe model behavior, not causal effects, and risk strata require external and prospective evaluation before individual-level use.

## Conclusions

Older adults moved frequently between functional and household financial vulnerability states, and dual vulnerability carried a distinctly adverse prognosis. A transparent penalized model provided moderate discrimination for dual vulnerability and death in the complete 2015–2018 temporal evaluation, while isolated CHE remained difficult to forecast. Exploratory 2020 analyses supported the observable mortality and ADL components only. The model may support coordinated geriatric, rehabilitation, and financial-protection review, but external validation, improved financing variables, and prospective impact assessment remain necessary.

## Supplementary Material

Supplementary Files

This is a list of supplementary files associated with this preprint. Click to download.

- Additionalfile1Supplementarymethodsresults.docx
- Additionalfile2TRIPODAIchecklist.docx
- Additionalfile3Reproducibility.zip

## Figures and Tables

**Figure 1:**
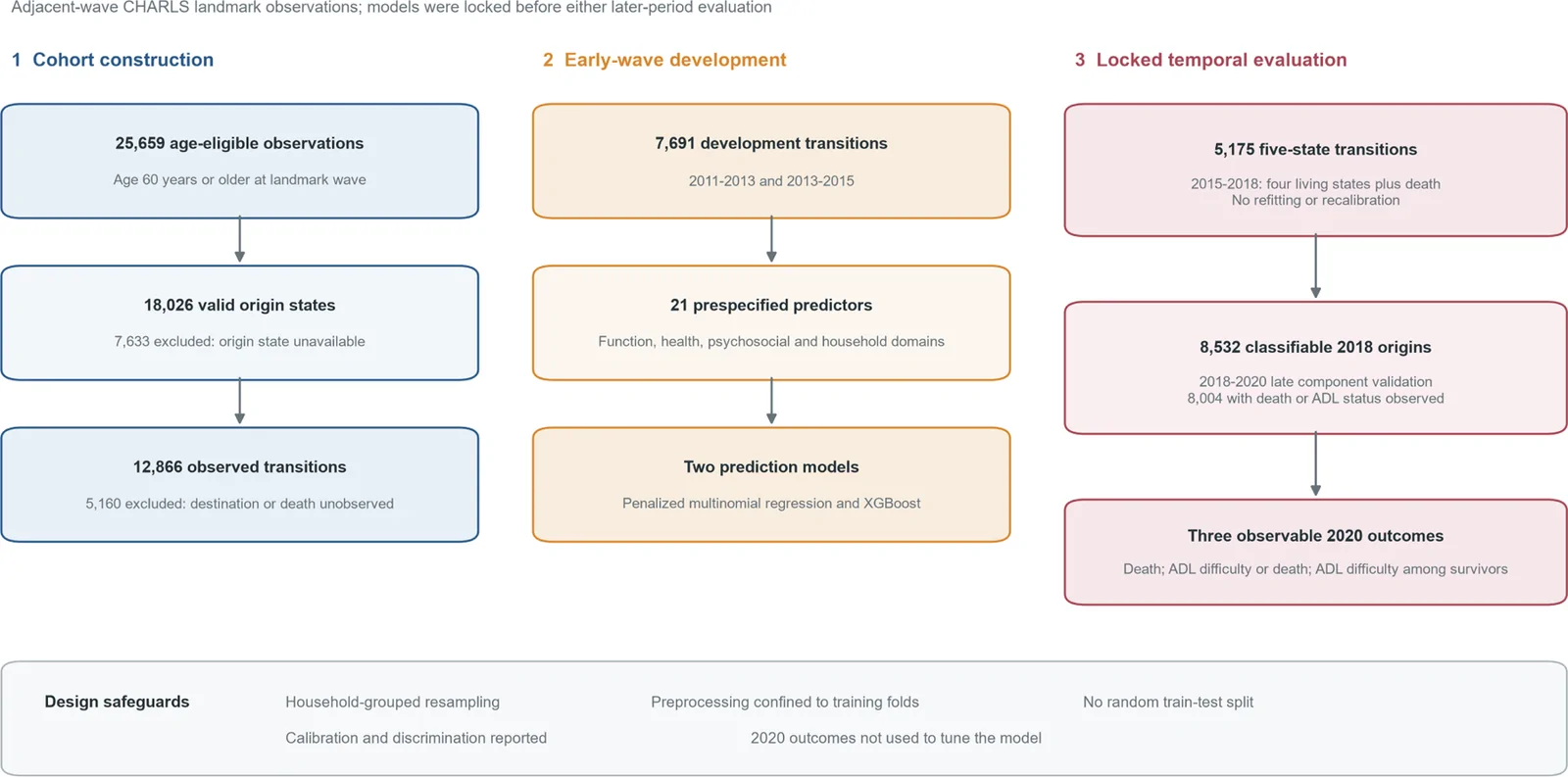
Study design, cohort construction, and evaluation sequence

**Figure 2:**
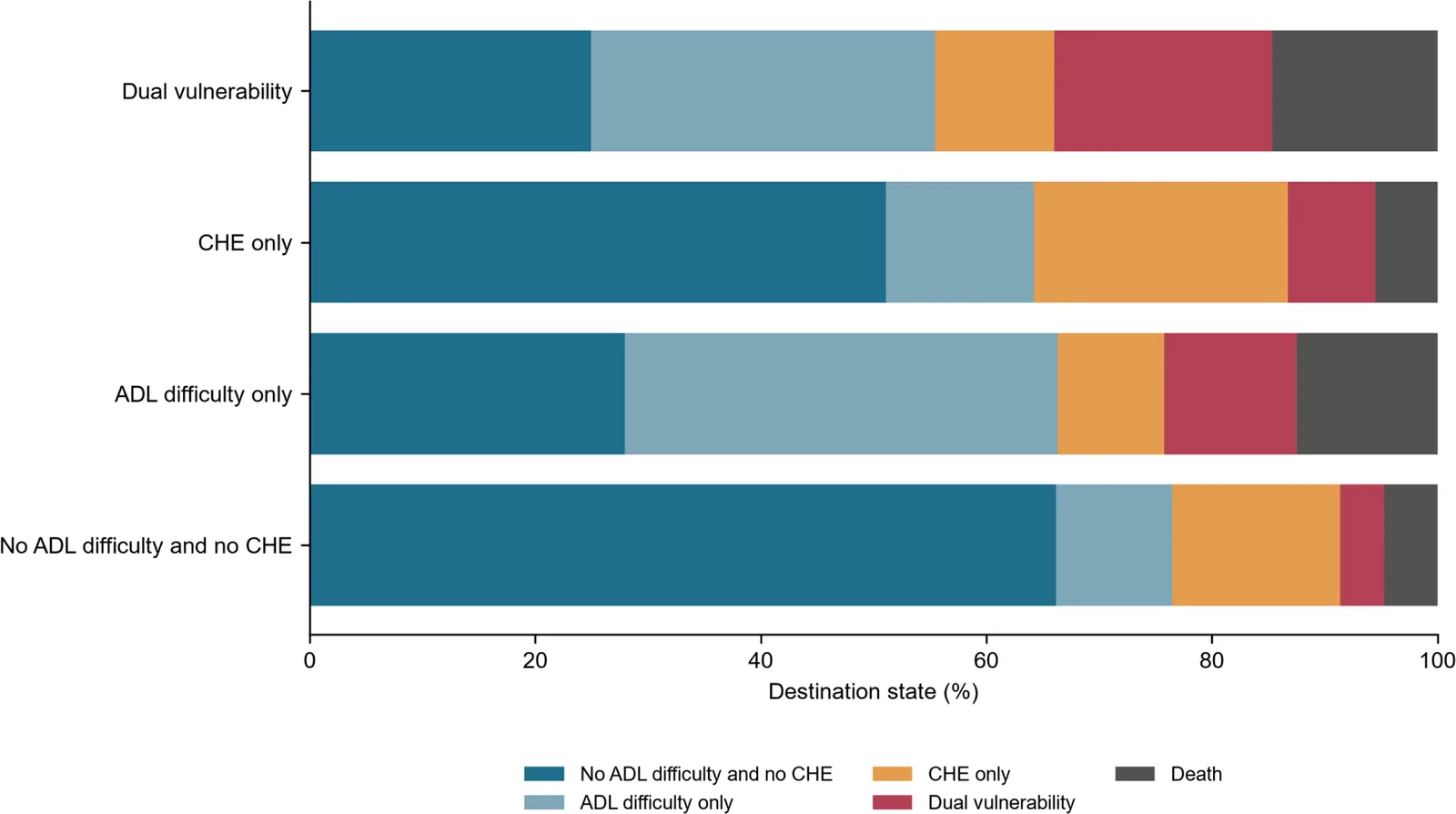
Observed destination states by origin state

**Figure 3:**
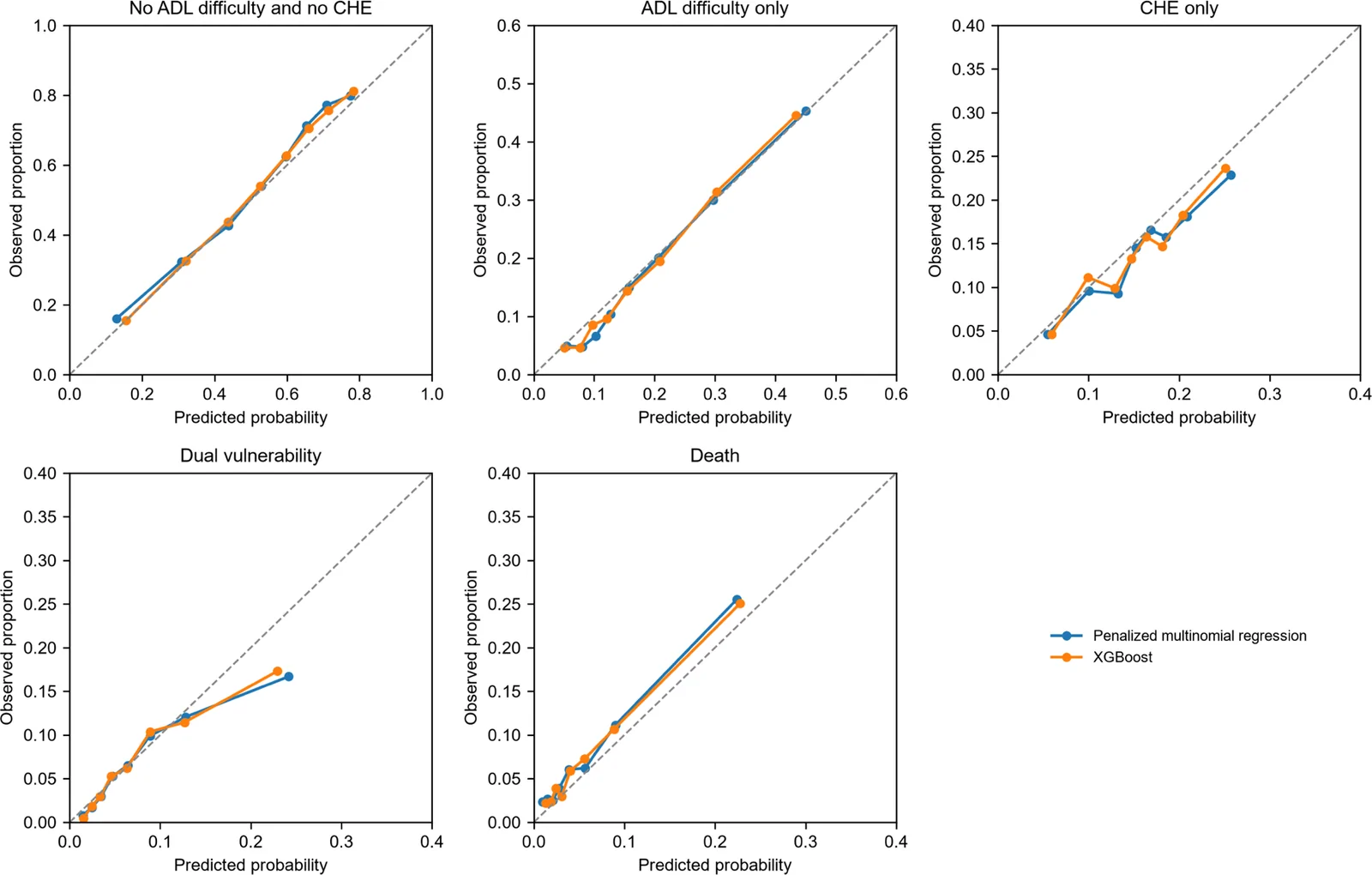
Calibration in the temporal evaluation

**Figure 4:**
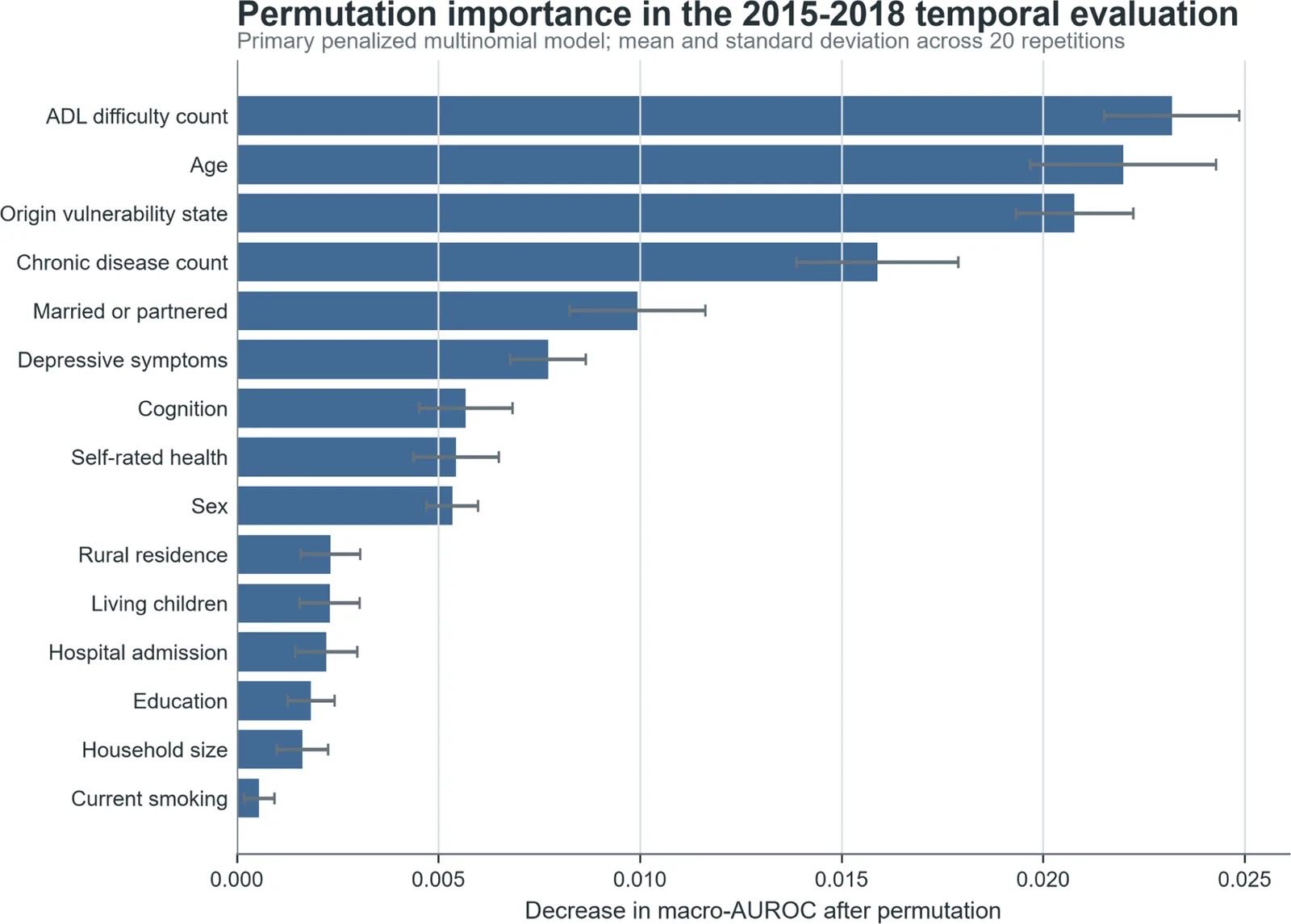
Permutation importance in temporal evaluation

**Figure 5:**
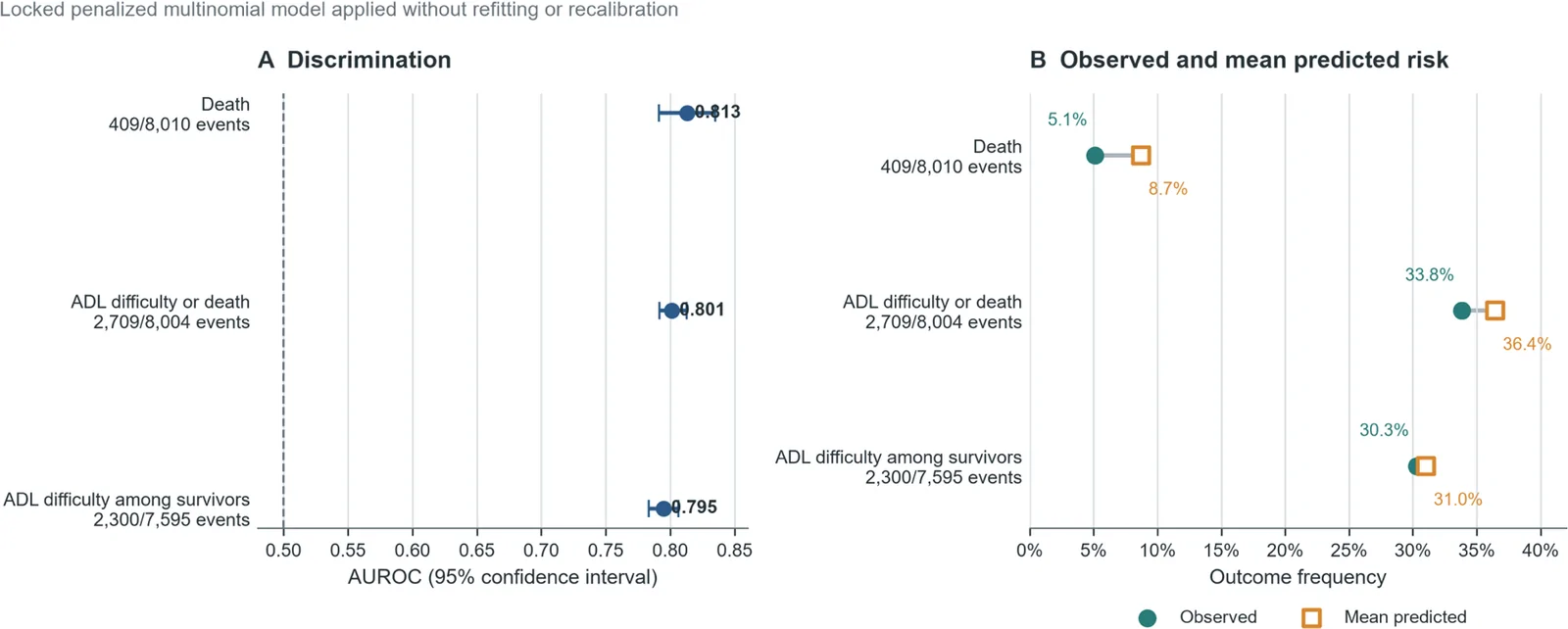
Late temporal component validation in 2018–2020

**Figure 6:**
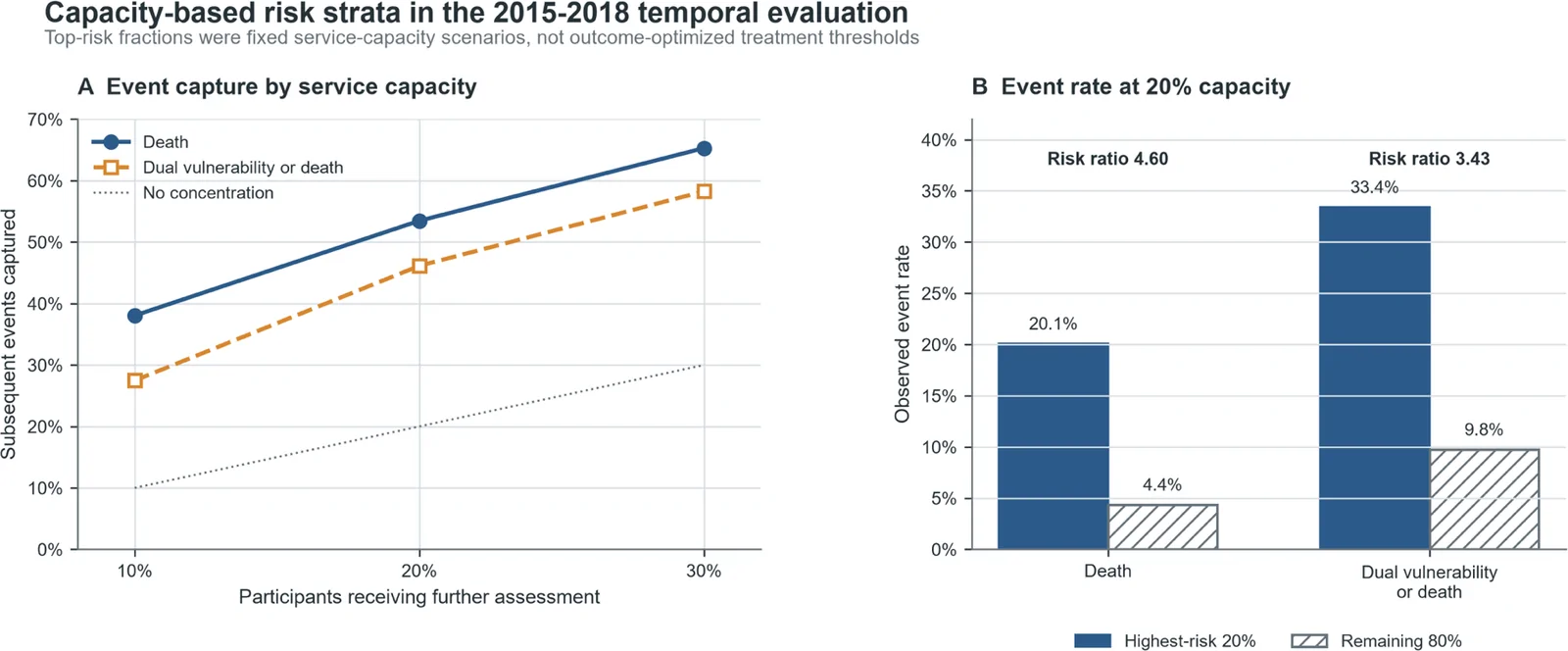
Capacity-based risk strata in temporal evaluation

**Table 1:** First eligible landmark characteristics

Characteristic	Value (N = 7,353)	Missing, n
Age, years	66.42 (6.42)	0
Women	3,543 (48.2%)	0
No formal education/did not finish primary	3,962 (53.9%)	1
Sishu or elementary school	1,850 (25.2%)	1
Middle school or higher	1,540 (20.9%)	1
Married/partnered	6,148 (83.6%)	0
Rural residence	4,728 (64.3%)	0
Number of ADL difficulties	0.53 (1.20)	0
Number of chronic conditions	1.60 (1.38)	320
CESD-10 score	8.67 (6.40)	366
Cognitive score	12.76 (5.39)	826
Household size	3.14 (1.74)	0
Number of living children	3.17 (1.55)	0
Public health insurance	6,576 (89.4%)	242
Social activity	3,245 (44.1%)	310
Hospital admission in past year	968 (13.2%)	0
Outpatient visit in past month	1,356 (18.4%)	1
Origin: no ADL difficulty and no CHE	4,533 (61.6%)	0
Origin: ADL difficulty only	1,302 (17.7%)	0
Origin: CHE only	1,027 (14.0%)	0
Origin: dual vulnerability	491 (6.7%)	0

**Table 2:** Primary penalized model performance in temporal evaluation

Destination state	Events	AUROC (95% CI)	AUPRC (95% CI)	Brier	Mean predicted	Calibration slope
No ADL difficulty and no CHE	2,818 (54.5%)	0.746 (0.732–0.758)	0.747 (0.726–0.768)	0.203	51.7%	1.035
ADL difficulty only	887 (17.1%)	0.753 (0.734–0.773)	0.383 (0.355–0.419)	0.124	18.4%	1.134
CHE only	720 (13.9%)	0.629 (0.605–0.650)	0.201 (0.179–0.227)	0.117	15.8%	0.972
Dual vulnerability	361 (7.0%)	0.728 (0.701–0.753)	0.153 (0.127–0.183)	0.063	8.0%	0.847
Death	389 (7.5%)	0.755 (0.727–0.781)	0.268 (0.230–0.320)	0.063	6.0%	0.906

**Table 3:** Overall temporal performance by model

Model	Macro-AUROC (95% CI)	Macro-AUPRC (95% CI)	Multiclass Brier (95% CI)	Log loss
Penalized multinomial regression	0.722 (0.712–0.734)	0.351 (0.340–0.366)	0.570 (0.557–0.583)	1.137
XGBoost	0.720	0.350	0.569	1.134

**Table 4:** Late temporal component validation in 2018–2020

Outcome	Events/N	AUROC (95% CI)	AUPRC (95% CI)	Brier	Observed/predicted	Calibration slope
Death	409/8,010	0.813 (0.791–0.835)	0.272 (0.231–0.324)	0.045	5.1%/8.7%	0.978
ADL difficulty or death	2,709/8,004	0.801 (0.792–0.813)	0.697 (0.682–0.716)	0.164	33.8%/36.4%	0.998
ADL difficulty among survivors	2,300/7,595	0.795 (0.783–0.807)	0.648 (0.623–0.670)	0.159	30.3%/31.0%	1.016

## Data Availability

The CHARLS data analyzed in this study are available to registered researchers from the CHARLS data portal (https://charls.charlsdata.com/pages/data/111/en.html). Harmonized CHARLS Version D data and documentation are available from the CHARLS Harmonized Data page (https://charls.pku.edu.cn/sj/Harmonized_CHARLS.htm). Data use is subject to the CHARLS user agreement; the authors cannot redistribute respondent-level files. Analysis code, the data-file checksum, and aggregate output tables are included with this submission as Additional file 3.

## Abbreviations

ADL: activities of daily living
AUPRC: area under the precision–recall curve
AUROC: area under the receiver operating characteristic curve
CHE: catastrophic health expenditure
CHARLS: China Health and Retirement Longitudinal Study
CESD-10: 10-item Center for Epidemiologic Studies Depression scale
OOP: out-of-pocket
TRIPOD + AI: Transparent Reporting of a multivariable prediction model for Individual Prognosis Or Diagnosis plus Artificial Intelligence
XGBoost: extreme gradient boosting
